# A Strong Graded Relationship between Level of Obesity and COPD: Findings from a National Population-Based Study of Lifelong Nonsmokers

**DOI:** 10.1155/2018/6149263

**Published:** 2018-11-20

**Authors:** Esme Fuller-Thomson, Kaitlyn E. N. Howden, Lilia R. Fuller-Thomson, Senyo Agbeyaka

**Affiliations:** ^1^Factor-Inwentash Faculty of Social Work and Department of Family and Community Medicine, Director, Institute of Life Course & Aging, University of Toronto, 246 Bloor Street West, Toronto, Ontario, Canada M5S 1A1; ^2^McMaster University, Michael G. DeGroote School of Medicine, 1280 Main Street West, Michael DeGroote Centre for Learning and Discovery, Hamilton, Canada ON L8S 4K1; ^3^Institute of Life Course & Aging, University of Toronto, 246 Bloor St. W., Room 238, Toronto, Ontario, Canada M5S 1V4; ^4^Factor-Inwentash Faculty of Social Work and University Health Network, 246 Bloor St. W., Room 238, Toronto, Ontario, Canada M5S 1V4

## Abstract

Factors associated with chronic obstructive pulmonary disease (COPD) among never-smokers have received little research attention. One potential risk factor for COPD is obesity, which is of particular importance in light of the global obesity epidemic. The objective of this study was to investigate the association between COPD and levels of obesity in a nationally representative sample of non-Hispanic white never-smokers. Data were drawn from the 2012 Center for Disease Control's Behavioral Risk Factor Surveillance System (BRFSS). Pearson's chi-square tests and logistic regression analyses were conducted in a large nationally representative sample of non-Hispanic white respondents aged 50 and over (76,004 women; 37,618 men) who reported that they had never smoked. A dose-response relationship was observed for both men and women: the prevalence of COPD increased from 2.5% in men and 3.5% in women who were of a healthy weight (BMI < 25) to 7.6% in men and 13.4% in women who had a BMI of 40 or higher. Even after adjusting for 7 potential confounds (e.g., age, education, and income), the odds of COPD were 3.21 higher for men (95% CI = 2.46, 4.20) and 4.00 higher for women with class III obesity (95% CI = 3.52, 4.55) in comparison with those of healthy weight. Regular screening for COPD is warranted in never-smoking obese patients who are aged 50 and over. Future research is needed to investigate plausible mechanisms for this association, including (1) the role of chronic inflammation associated with obesity and (2) the impact of central obesity on respiratory system mechanics.

## 1. Introduction

Chronic obstructive pulmonary disease (COPD) is a major public health issue. Fifteen million Americans are diagnosed with COPD [1] with estimates varying in each state from 4% to 9% of the population [2]. Approximately 135,000 Americans die from COPD each year, making it the third leading cause of death in the United States [2]. In recent years, mortality rates due to COPD have shown a significant increase, particularly among women [3]. COPD is one of the major causes of disability [4], impacting individuals' ability to work, sleep, and participate in household chores and family activities [2]. In 2010, 50 billion dollars annually was spent on COPD, approximately 60% of which was due to direct health expenditures [2]. The remaining 40% of the costs were due to lost productivity because of illness and premature mortality [2]. Although smoking is the best-known risk factor for COPD, 23% of those with the condition have never smoked [5].

New research suggests that the association between obesity and COPD warrants greater research attention [6]. Obesity in adults is defined as having a body mass index (BMI) of 30 or higher [7]. The World Health Organization further divides obesity into class I (BMI 30–34.99), class II (BMI 35–39.99), and class III (BMI of 40 or higher) [8]. Individuals that fall into the class III category tend to be 100–200 pounds (45–90 kg) overweight [9]. One in every 16 Americans (6.2%) has class III obesity [10]. From 2000 to 2005, the prevalence of a BMI over 40 increased by 50% [11]. This rapid increase is particularly concerning to the healthcare system because individuals with class III obesity encounter more serious health problems than those with moderate obesity and have much higher annual healthcare costs [11].

Obesity is a major health risk that is linked to several life-threatening diseases, including stroke, type 2 diabetes, hypertension, coronary artery disease, and a number of different cancers [12]. The United States has one of the highest rates of obesity in the world, with more than one-third of US adults classified as obese [13]. This growing phenomenon is concerning, with obesity now ranking as the seventh leading cause of death in the United States [14], with approximately 300,000 deaths directly linked to obesity each year [15]. Furthermore, people with obesity have medical costs 30% higher than those of a healthy weight [16]. In 2008, obesity-related medical costs had risen to $75 billion dollars, with projections suggesting they will comprise 16–18% of the total US healthcare expenditure by 2030 [16].

Cross-sectional studies have demonstrated an inverse association between obesity and the prevalence of COPD [6]. Many of these studies included both never-smoking and ever-smoking samples, were not sex specific, and failed to adjust statistically for other known risks for COPD. The direction of the association is unclear. It is possible that improper lung function increases the risk of developing obesity. This may be due to the following three causes: (1) COPD patients' difficulty breathing while exercising often results in lower levels of physical activity and thus fewer calories burned in exercise, (2) a common side effect of long-term glucocorticosteroid medications is weight gain [6], and (3) due to being hypoxemic both at rest and with exercise, COPD patients are unable to utilize oxygen for the breakdown of fatty acids through beta oxidation. Without this pathway, their bodies are unable to properly utilize fat as an energy source during bouts of aerobic exercise and their cells are forced to utilize less efficient means of energy production via anaerobic pathways [17]. However, a recent prospective study of almost 50,000 American Association of Retired Persons (AARP) aged 50 to 70 who had never smoked provides important support for the hypothesis that obesity puts individuals at a greater risk for COPD [18]. In their analysis, never-smoking adults with a BMI of 35 or more without COPD, cancer, or heart disease in 1995 had 36% higher odds of developing COPD by 2006, and that relationship remained after adjusting for socio-demographics, height, diabetes, and alcohol intake at baseline [18].

Although the association between obesity and COPD is increasingly recognized, the mechanisms involved and the nature of the relationship are still unclear. One reason for this uncertainty is that studies looking at COPD typically have included current smokers or those with a history of smoking (e.g., [19]). Since smoking is the number one risk factor for the development of COPD [20], including smokers in the studies makes it more difficult to investigate whether obesity is playing an independent role in the development of COPD, aside from the smoking, and whether obesity is associated with a dose-response relationship.

The purpose of this paper is to determine if there is an association between obesity level and prevalence of COPD. Since there are marked sex and race differences in vulnerability to COPD among never-smokers, and there is an interaction between sex and race [21], it is important to conduct sex specific analyses and to focus on one particular race [22]. For the purposes of this study, we have focused on the largest single racial group, non-Hispanic white individuals. The data set we used, the Behavioral Risk Factor Surveillance System (BRFSS), did not contain information on immigration status. In the developing world, indoor use of biomass fuels such as charcoal and wood for cooking is a major cause of COPD among never-smokers [23]. To minimize the likelihood of this confound, we chose to include only non-Hispanic whites in this study, because the majority of non-Hispanic white immigrants came from countries in Europe, as well as Australia and Canada, where use of biomass for cooking and heating is unusual. Socioeconomic status (SES), measured by both education and income levels, is inversely associated with both obesity [24] and elevated levels of COPD [25] and thus must be taken into account. A review of literature on the effect of socioeconomic status on COPD suggests that the association between the two is multifactorial in nature with housing conditions, exposure to toxins during prenatal and childhood, diet, and lifestyle choices contributing to the association [25].

## 2. Methods

### 2.1. Data Source and Sample

As described elsewhere [26], the data were derived from the CDC's 2012 BRFSS [27], a nationally representative telephone survey of the health and health behaviors of noninstitutionalized adults. Due to the long latency of COPD, the sample was restricted to respondents aged 50 and older who had never smoked. Current or former smokers were excluded in order to investigate the association between obesity and COPD without confounding by smoking, the largest known risk factor for the disease. As was discussed above, the sample was restricted to non-Hispanic white respondents because we did not have information in the BRFSS on immigration status and we wanted to minimize the probability that respondents had been immigrants from the developing world where indoor cooking with charcoal or wood contributes substantially to the incidence of COPD among never-smokers [23]. To minimize the likelihood of this confound, we chose to include only non-Hispanic whites in this study because the majority of these immigrants would come from countries in Europe, as well as Australia and Canada, where use of biomass for cooking is unusual. The final unweighted sample included 76,004 women and 37,618 men, of whom 2,895 women and 1,069 men had class III obesity and 4,048 women and 1,153 men had COPD.

### 2.2. Measures

#### 2.2.1. Outcome

COPD was dichotomized based on participants' response to a question if they had been told by a doctor, nurse, or other health professionals that they had “chronic obstructive pulmonary disease or COPD, emphysema, or chronic bronchitis?”

#### 2.2.2. Exposure of Interest

*Obesity:* Self‐reported weight in kilograms (kg) was divided by self-reported height in squared metres (m^2^) to define BMI, which was then categorized into ranges defining normal weight (BMI < 25 kg/m^2^), overweight (BMI = 25–29.99 kg/m^2^), obese class 1 and 2 (BMI = 30 kg/m^2^ to 39.99), and class III obesity (BMI = 40 or higher).

#### 2.2.3. Control Variables

Several control characteristics were assessed, including sex, age (categorized by decade: 50s, 60s, 70s, and 80+), and self-reported height in inches. Education was assessed based on the following categories: did not graduate high school, graduated high school, attended college or technical school, and graduated from college or technical school. Household income was categorized as less than $15,000, $15,000–$24,999, $25,000–$ 49,999, $50,000–$74,999, $75,000 or above, and a missing category.

Second-hand smoke exposure is also a substantial risk factor for COPD among never-smokers, with an estimated 9% of cases due to exposure to second-hand smoke at home [28]. Although we did not have a direct measure of such exposure, we assumed never married respondents and those who lived alone would be the least likely to have been exposed to second-hand smoke at home. Marital status was dichotomized as having ever been married or currently living common-law versus never married. The number of individuals in the household was categorized as one, two, three or more, and missing.

Since we relied upon self-report of a medical diagnosis of COPD, we were concerned that those without health coverage may be less likely to have been assessed for COPD. To address this potential bias, we included healthcare coverage based upon the question “Do you have any kind of healthcare coverage, including health insurance, prepaid plans such as HMOs, or government plans such as Medicare, or Indian Health Service?”

### 2.3. Statistical Analyses

Bivariate analyses using Pearson's chi-square tests were conducted to assess the prevalence of COPD by obesity and each of the control variables. Of particular interest was the degree to which potential confounds might attenuate the relationship between obesity and COPD. To determine if we needed sex specific analyses, we conducted a logistic regression analysis testing for interaction effects between sex and obesity level (analyses not shown). The interaction effect was statistically significant (*p* < 0.001). Thus, logistic regression analyses were conducted separately for men and women, with obesity level as the focal exposure and COPD as the outcome variable. In the first logistic regression, only obesity level was in the model. In the second logistic regression, all of the control variables were added to the analysis. Data were weighted to correct for nonresponse and likelihood of selection.

## 3. Results

Among non-Hispanic white women aged 50 and over who were lifelong nonsmokers, the prevalence of COPD increased in a graded fashion from 3.5% in women who were of normal weight (BMI < 25) to 13.4% in women who had class III obesity (BMI of 40 or higher; *p* < 0.001) (Table 1). Even after adjusting for all 7 potential confounds, the odds of COPD among women were 25% higher for those in the overweight category (95% CI = 1.15, 1.37), twice as high for those in class I or II obesity (OR = 2,12; 95% CI = 1.94, 2.31), and four times higher (95% CI = 3.52, 4.55) for women who had class III obesity in comparison with those with normal weight (Table 2).

Among men, the prevalence of COPD was similar for those in the normal weight and overweight category (2.5% and 2.2%, respectively), but was substantially higher in those with class I and II obesity (3.8%) as well as those with class III obesity (7.6%) (*p* < 0.001) (Table 3). As is shown in Table 4, after adjustments for 7 potential confounds, the odds of COPD among men were 71% higher for men with class I or II obesity (OR=1.71; 95% CI=1.43, 2.04) and 3-fold higher for men with class III obesity (OR = 3.21; 95% CI = 2.46, 4.20) in comparison with men who were normal weight. Men in the overweight but not obese category (i.e., BMI > 24.99 and BMI < 30) did not differ from those in the normal weight category in the logistic regression analyses.

Among both women and men, the odds of COPD were higher among older respondents and those with lower income. Among women, but not men, height and education level were negatively associated with COPD, and being married was associated with higher odds of COPD. Among men, but not women, being without a health plan was associated with lower odds of reporting a COPD diagnosis. The number of current housemates were not associated with COPD among men or women.

## 4. Discussion

There was a strong, graded relationship between COPD and obesity level for both men and women, and this relationship was not explained by many of the known risk factors for COPD such as age, income, and education. The prevalence of COPD among never-smoking non-Hispanic white Americans aged 50 and over with class III obesity is very high with one in every 7 never-smoking women with class III obesity and one in every 13 men with class III obesity reporting that they have been diagnosed with COPD.

We had anticipated that controlling for SES would substantially attenuate the association between obesity and COPD, due to the fact that SES is strongly associated with both factors [29, 30]. Previous research indicates that individuals with a lower income have a significantly higher prevalence of exposure to second-hand smoke and air pollution than the rest of the population [31, 32]. Between 2011 and 2012, 25.3% of the total population was exposed to second-hand smoke while the rates increase to 43.2% for people living below the poverty level [32]. A study controlling for smoking history concluded that approximately 1 in 11 COPD cases are caused by exposure to second-hand smoke at home, and 1 in 15 are the result of exposure to second-hand smoke in the workplace [28]. Although we found a steep inverse gradient between income level and COPD, adjusting for income and all the other control variables only resulted in an extremely modest change for either women or men in the odds ratios associated with COPD among those with class III obesity in comparison with those with BMI <25.

A higher percentage of women are exposed to second-hand smoke and air pollution given they are more likely to live below the poverty line in comparison with men [31] and, given the historically higher prevalence of smoking among men, married nonsmoking women are more likely to have been exposed to second-hand smoke than married nonsmoking men. Our finding are in keeping with this, as we found that marital status was significantly associated with COPD for women in our study, but not for men.

There are three plausible explanations for the strong dose-response association we found between obesity level and COPD in both men and women: (1) obesity causes chest wall compression and impairs diaphragm movement; (2) inflammation associated with obesity causes or exacerbates COPD; and (3) the symptoms of COPD result in decreased mobility and energy expenditure that concomitantly increases BMI. Obesity, particularly central obesity, has been linked to many respiratory problems, including COPD [33]. Deleterious changes in lung function due to excess weight are not limited to those with class III obesity as they can occur even in overweight individuals [33]. Obesity results in greater demand on the lungs because the larger body mass consumes more oxygen and obesity impairs exercise capacity, often leading to more sedentary lives [34]. Fat build-up localized to areas such as the anterior chest wall, anterior abdominal wall, and visceral organs can lead to decreased diaphragm mobility and lowered chest wall compliance, which then increases the effort needed to breathe [33, 35].

Inflammation may also play a role in the obesity-COPD association. Obese individuals have elevated levels of a variety of inflammatory markers, including TNF-a, IL-6, and adipose resident macrophages, which results in an increased level of inflammation both locally and systemically [36]. Inflammation may be connected to developing or exacerbating COPD due to the role it can play in impairing lung function. Increased inflammation can lead to airway remodeling, including progressive narrowing of the small airways due to fibrosis, increased mucus production due to more goblet cells, and alveolar wall destruction [37]. This reaction is typically due to a maladaptive response from the body to environmental stimuli, particularly those associated with cigarette smoking [38]. However, increased activation and function of these inflammatory cells can still be observed in those who have never smoked [39]. The reason for this phenomenon could be due to the equally important role that systemic inflammation plays in the development of this disease. Patients with COPD also have higher levels of proinflammatory cytokines in circulation, and studies such as the Framingham Heart Study have found that the level of systemic inflammatory biomarkers is correlated with impaired lung function and the development of COPD [38]. The Framingham study examined the systemic inflammatory markers in individuals who smoked, but it is plausible that the adipose-induced inflammation in never-smokers with class III obesity may be causing the same effect [40].

The previous two hypotheses suggest that obesity may cause or exacerbate COPD. Due to the cross-sectional nature of the current study, we cannot determine the direction of the association. An alternate hypothesis, suggesting reverse-causality, is that the symptoms of COPD may impede mobility to the point of decreasing energy expenditure which, if food consumption remains constant, could result in increasing weight. If that is the case, then one would expect patients with the most severe COPD symptoms would have the most limited mobility and thus be the heaviest. However, the opposite is true. Approximately half of those with severe COPD experience weight loss in comparison with one in every seven or ten patients with mild-to-moderate COPD [41].

It would be interesting for future research to explore if alpha-1 antitrypsin (AAT) deficiency plays any role in the link between obesity and COPD among those who do not smoke. AAT is a protein that is sensitive to inflammation [42]. It protects the lung tissue from neutrophil elastase, stopping it from breaking down elastin [43]. A large cross-sectional study in the general population found that BMI was inversely associated with levels of alpha-1 antitrypsin [44]. AAT deficiency is an inherited disorder that puts individuals at considerable risk for deterioration in lung function and COPD in those with and without a history of smoking [45].

There are limitations to this study that must be considered when interpreting results. One major concern is that the identification of those with COPD was based upon self-report of a medical diagnosis of COPD. Previous research has indicated that there is substantial agreement between those who report they have a medical diagnosis of COPD and chart review, ranging from 86% [46] to 97% concordance [47]. However, many of those who report they do not have a medical diagnosis actually do have the disease, but have not been tested and are therefore unaware they have COPD. This error is particularly common for those with mild symptoms [48]. In support of this hypothesis, we found that men without health insurance, who we surmise may have less regular access to healthcare and thus fewer opportunities of being diagnosed than those with insurance, had 28% lower odds of COPD. There was no association for women. However, adjusting for health plan coverage did not substantially attenuate the direct association between obesity and COPD in either women or men. If underreporting of COPD did occur at random, this would result in a bias toward the null, making our results more conservative. Without the benefit of chart review, we do not know if the respondents were diagnosed only based upon a low FEV1 level measured in a physician's office or on a full work-up of many pulmonary function tests (PFT) in a hospital or laboratory setting. The latter is the gold standard for COPD diagnosis. Some people with obesity who are diagnosed with COPD only based upon lower FEV1 levels may in fact not have an obstructive disease but instead have a restrictive respiratory pattern due to excess fat in the abdomen and chest constraining expiration volume [49]. In future research, it would be beneficial to have all patients diagnosed through a complete battery of pulmonary function tests.

We also relied upon self-report of weight and height to calculate BMI level. Research indicates self-reported information tends to underestimate obesity compared to actual measurements, particularly among women and heavier respondents [50], although such an underreporting would bias our findings toward the null. We are missing important information on known risk factors for COPD among never-smokers such as exposure to biomass fuel for cooking and exposure to second-hand smoke. We attempted to partially address these limitations by restricting our sample to non-Hispanic whites and controlling for marital history and number of housemates, respectively. Even with these adjustments, a graded association existed between COPD and level of obesity.

A major limitation is the cross-sectional nature of the data, which could only determine association, not causation. Future research would benefit from prospective large, nationally representative samples of never-smoking older adults who are free of COPD at baseline, in order to determine if level of obesity predicts COPD onset.

Despite these limitations, this study provides important population-based findings in older, never-smokers documenting a robust dose-response association between obesity level and COPD in both sexes. As the percentage of never-smokers in the older population increases, concomitant with an epidemic of obesity, our findings of a strong obesity-COPD relation may be of growing relevance to public health in coming years. If this link is validated and the association is found to be causal in future prospective research, health professionals should be careful not to overlook COPD screening and treatment in their never-smoking patients, particularly for those with class III obesity and those with alpha-1 antitrypsin deficiency. Clinicians who treat patients with both obesity and COPD should help patients develop effective weight loss strategies. In addition to referring to a dietician to promote calorie reduction, referral to outpatient pulmonary rehabilitation therapy may be helpful. A specialized rehabilitation program is warranted for those with exercise-induced hypoxemia and obesity to develop a therapeutic exercise plan with appropriate use of supplemental oxygen therapy.

## Figures and Tables

**Table 1:** Prevalence of COPD by Obesity Level and other Characteristics among non-Hispanic white older women (aged 50+) who had never smoked (*n*=76,004)^1^.

	No COPD	COPD	*p* value^2^
	*n*=71,956 (94.7%)	*n*=4,048 (5.3%)	
Obesity			
** **BMI < 25 (ref)	96.5%	3.5%	<0.001
** **Overweight (BMI = 25–29)	95.4%	4.6%	
** **Obese (BMI = 30–39.9)	92.4%	7.6%	
** **Class III obesity (BMI ≥ 40)	86.6%	13.4%	

Age by decade			
** **50s (ref)	96.5%	3.5%	<0.001
** **60s	94.6%	5.4%	
** **70s	93.5%	6.5%	
** **80+	92.9%	7.1%	
Height (mean, SD)	64.1 (2.7)	63.6 (2.8)	<0.001

Socioeconomic status			
** **Education			
** **Did not graduate high school	91.0%	9.0%	<0.001
** **Graduated high school	94.1%	5.9%	
** **Attended college or technical school	94.7%	5.3%	
** **Graduated from college or technical school	96.9%	3.1%	
** **Household income			
** **$75,000 or more (ref)	97.8%	2.2%	<0.001
** **$50,000 to less than $75,000	96.3%	3.7%	
** **$25,000–$49,999	94.1%	5.9%	
** **$15,000–$24,999	92.3%	7.7%	
** **<$15,000	88.3%	11.7%	
** **Missing	94.7%	5.3%	

Household conditions			
** **Marital status			
** **Married/common-law	94.8%	5.2%	=0.208
** **Never married	95.3%	4.7%	
** **Number of adults/households			
** **1	93.4%	6.6%	<0.001
** **2	95.6%	4.4%	
** **≥3	95.3%	4.7%	
** **Missing	94.2%	5.8%	

Health plan			
** **Yes (ref)	94.9%	5.1%	<0.001
** **No	93.3%	6.7%	

^1^Sample sizes are presented in their unweighted form. Percentages are weighted to adjust for the probability of selection and nonresponse. ^2^ *p* value is derived from a chi-square test for categorical variables and *t*-test for continuous variables. Source: Behavioral Risk Factor Surveillance System 2012. SD = standard deviation.

**Table 2:** Logistic regression analyses of COPD among non-Hispanic white older women (aged 50+) who had never smoked (*n*=76,004) (odds ratios and 95% confidence intervals (CIs)).

	Model 1: obesity	Model 1: 95% CI	Model 2: fully adjusted	Model 2: 95% CI
Obesity				
** **BMI < 25 (ref)	1.00		1.00	
** **Overweight (BMI = 25–29)	1.33	(1.23, 1.45)	1.26	(1.15, 1.37)
** **Obese (BMI = 30–39.9)	2.26	(2.08, 2.46)	2.13	(1.96, 2.32)
** **Class III obesity (BMI ≥ 40)	4.29	(3.80, 4.85)	4.02	(3.54, 4.58)

Age by decade				
** **50s (ref)			1.00	
** **60s	—		1.37	(1.25, 1.50)
** **70s	—		1.55	(1.40, 1.72)
** **80+	—		1.74	(1.55, 1.95)
Height	—		0.99	(0.97, 1.00)

Socioeconomic status				
** **Education				
** **Did not graduate high school	—		1.33	(1.17, 1.52)
** **Graduated high school	—		1.16	(1.05, 1.28)
** **Attended college or technical school	—		1.25	(1.13, 1.38)
** **College or technical school graduate (ref)	—		1.00	
** **Household income				
** **$75,000 or more (ref)	—		1.00	
** **$50,000 to less than $75,000	—		1.47	(1.27, 1.69)
** **$25,000–$49,999	—		2.09	(1.84, 2.36)
** **$15,000–$24,999	—		2.47	(2.15, 2.82)
** **<$15,000	—		3.76	(3.24, 4.37)
** **Missing	—		1.94	(1.70, 2.22)

Household conditions				
** **Marital status				
** **Married at least once	—		1.28	(1.09, 1.50)
** **Never married (ref)	—		1.00	
** **Number of adults/households				
** **1 (ref)	—		1.00	
** **2	—		0.92	(0.85, 1.00)
** **≥3	—		1.06	(0.95, 1.18)
** **Missing	—		1.18	(1.05, 1.33)

Health plan				
** **Yes (ref)	—		1.00	
** **No	—		1.09	(0.95, 1.26)
−2 log likelihood	30315.5		29299.2	

**Table 3:** Prevalence of COPD by obesity level and other characteristics among non-Hispanic white older men (aged 50+) who had never smoked (*n*=37,618)^1^.

	No COPD	COPD	*p* value^2^
	*n*=36,465 (96.9%)	*n*=1,153 (3.1%)	
Obesity			
** **BMI < 25 (ref)	97.5%	2.5%	<0.001
** **Overweight (BMI = 25–29)	97.8%	2.2%	
** **Obese (BMI = 30–39.9)	96.2%	3.8%	
** **Class III obesity (BMI ≥ 40)	92.4%	7.6%	

Age by decade			
** **50s (ref)	98.1%	1.9%	<0.001
** **60s	96.8%	3.2%	
** **70s	95.5%	4.5%	
** **80+	95.9%	4.1%	
Height (mean, SD)	70.3 (2.8)	69.3 (3.2)	<0.001

Socioeconomic status			
** **Education		55.59%	
** **Did not graduate high school	94.1%	5.9%	<0.001
** **Graduated high school	96.7%	3.3%	
** **Attended college or technical school	96.9%	3.1%	
** **Graduated from college or technical school	97.9%	2.1%	
** **Household income			
** **$75,000 or more (ref)	98.6%	1.4%	<0.001
** **$50,000 to less than $75,000	97.9%	2.1%	
** **$25,000–$49,999	96.2%	3.8%	
** **$15,000–$24,999	94.4%	5.6%	
** **<$15,000	90.9%	9.1%	
** **Missing	96.8%	3.2%	

Household conditions			
** **Marital status			
** **Married/common-law	97.2%	2.8%	<0.001
** **Never married	96.0%	4.0%	
** **Number of adults/households			
** **1	95.5%	4.5%	<0.001
** **2	97.1%	2.9%	
** **≥3	97.5%	2.5%	
** **Missing	98.1%	1.9%	

Health plan			
** **Yes (ref)	97.0%	3.0%	0.773
** **No	97.1%	2.9%	

^1^Sample sizes are presented in their unweighted form. Percentages are weighted to adjust for the probability of selection and nonresponse. ^2^ *p* value is derived from a chi-square test for categorical variables and *t*-test for continuous variables. Source: Behavioral Risk Factor Surveillance System 2012. SD = standard deviation.

**Table 4:** Logistic regression analyses of COPD among non-Hispanic white older men (aged 50+) who had never smoked (*n*=37,618) (odds ratios and 95% confidence intervals (CIs).

	Model 1: obesity	Model 1: 95% CI	Model 2: fully adjusted	Model 2: 95% CI
Obesity				
** **BMI <25 (ref)	1.00		1.00	
** **Overweight (25–29)	0.91	(0.76, 1.07)	0.99	(0.83, 1.17)
** **Obese (BMI = 30–39.9)	1.55	(1.31, 1.84)	1.71	(1.43, 2.04)
** **Class III obesity (BMI ≥40)	3.25	(2.52, 4.20)	3.21	(2.46, 4.20)

Age by decade				
** **50s (ref)			1.00	
** **60s	—		1.53	(1.31, 1.79)
** **70s	—		1.95	(1.62, 1.34)
** **80+	—		1.61	(1.28, 2.03)
Height	—		0.99	(0.97, 1.01)

Socioeconomic status				
** **Education				
** **Did not graduate high school	—		1.20	(0.96, 1.53)
** **Graduated high school	—		0.97	(0.82, 1.15)
** **Attended college or technical school	—		1.09	(0.93, 1.29)
** **College or technical school graduate (ref)	—		1.00	
** **Household income				
** **$75,000 or more (ref)	—		1.00	
** **$50,000 to less than $75,000	—		1.32	(1.06, 1.66)
** **$25,000–$49,999	—		2.34	(1.93, 2.84)
** **$15,000–$24,999	—		3.48	(2.78, 4.35)
** **<$15,000	—		6.26	(4.86, 8.08)
** **Missing	—		2.01	(1.58, 2.56)

Household conditions				
** **Marital status				
** **Married at least once	—		0.94	(0.76, 1.17)
** **Never married (ref)	—		1.00	
** **Number of adults/households				
** **1 (ref)	—		1.00	
** **2	—		0.94	(0.79, 1.29)
** **≥3	—		0.95	(0.76, 1.19)
** **Missing	—		0.61	(0.48, 0.77)

Health plan				
** **Yes (ref)	—		1.00	
** **No	—		0.72	(0.56, 0.94)
−2 log likelihood	9694.55		9192.40	

## Data Availability

The data sets analyzed during the current study are available in the Centers for Disease Control and Prevention Behavioral Risk Factor Surveillance System repository (https://www.cdc.gov/brfss/annual_data/annual_2012.html).
